# A novel coronavirus capable of lethal human infections: an emerging picture

**DOI:** 10.1186/1743-422X-10-66

**Published:** 2013-02-28

**Authors:** Gulfaraz Khan

**Affiliations:** 1 Department of Microbiology and Immunology, College of Medicine and Health Sciences (Tawam Hospital Campus), United Arab Emirates University, PO Box 17666, Al Ain, United Arab Emirates

**Keywords:** Coronavirus (CoV), Severe respiratory infection, Renal failure

## Abstract

**Summary:**

In September 2012, a novel coronavirus was isolated from a patient in Saudi Arabia who had died of an acute respiratory illness and renal failure. The clinical presentation was reminiscent of the outbreak caused by the SARS-coronavirus (SARS-CoV) exactly ten years ago that resulted in over 8000 cases. Sequence analysis of the new virus revealed that it was indeed a member of the same genus as SARS-CoV. By mid-February 2013, 12 laboratory-confirmed cases had been reported with 6 fatalities. The first 9 cases were in individuals resident in the Middle East, while the most recent 3 cases were in family members resident in the UK. The index case in the UK family cluster had travel history to Pakistan and Saudi Arabia. Although the current evidence suggests that this virus is not highly transmissible among humans, there is a real danger that it may spread to other parts of the world. Here, a brief review of the events is provided to summarize the rapidly emerging picture of this new virus.

## The threat of respiratory viruses

Whenever a new virus associated with an acute respiratory illness emerges, medical authorities around the world are put on high alert and vigilance, and quite justifiably. Emerging and re-emerging viruses causing respiratory infections have been responsible for outbreaks resulting in millions of deaths. The pandemic influenza viruses of 1918, 1957 and 1968 are our bleak reminders. Since 1968, major outbreaks with mortalities on the scale previously seen have not occurred, although there have been regular threats.

## The novel coronavirus

Recently, a novel coronavirus has been identified in patients with severe acute respiratory illness [1,2]. This new virus, provisionally referred to as novel coronavirus (NCoV) has been fully sequenced and shown to belong to group C β-coronaviruses [3-5]. The genome, which is just over 30 KB, contains at least 10 predicted open reading frames (ORFs) [4]. The genome size, organization and sequence analysis revealed that the NCoV is most closely related to bat coronaviruses BtCoV-HKU4 and BtCoV-HKU5 [3-5] first isolated in 2006 from bats captured in Hong Kong [6]. The major difference between NCoV and these bat coronaviruses is in the region between the spike and the envelop genes [5]. The NCoV has 5 ORFs while the bat viruses have 4 in this region [5]. The nearest human coronavirus related to NCoV is SARS-CoV [3-5]. This virus was responsible for the outbreak of severe acute respiratory syndrome in 2002–2003 which resulted in 8,422 cases worldwide with 916 deaths [7]. With a mortality of approximately 11% seen with SARS-CoV infection, the identification of NCoV from patients with similar acute respiratory illness as with SARS-CoV is of a real concern. Coronaviruses are a large family of enveloped, single-stranded RNA viruses that infect a number of different species, including humans. They are usually species specific, however interspecies transmission of coronaviruses can occur [8-10]. Worryingly, *in vitro* studies show that NCoV is also capable of infecting cells from different species, including monkeys, humans, bats and pigs [2,11]. Indeed, NCoV was first isolated using monkey kidney epithelial cell lines, Vero and LLC-MK20, both of which are susceptible to infection and can propagate the virus relatively easily [2]. Prior to the isolation of NCoV, only five coronaviruses, namely 229E, OC43, SARS-CoV, HKU1 and NL63, were known to cause infections in humans [12]. In the absence of any underlying co-morbidities, all of these coronaviruses, except for SARS-CoV, are generally associated with mild upper respiratory tract infections. SARS-CoV has an unusual predilection for infecting cells in the lower respiratory tract. Although NCoV also causes lower respiratory tract infection, the viral receptor appears to be different from that used by SARS-CoV [11,13].

## Timeline for confirmed human cases (in chronological order of reporting)

**Figure 1:**
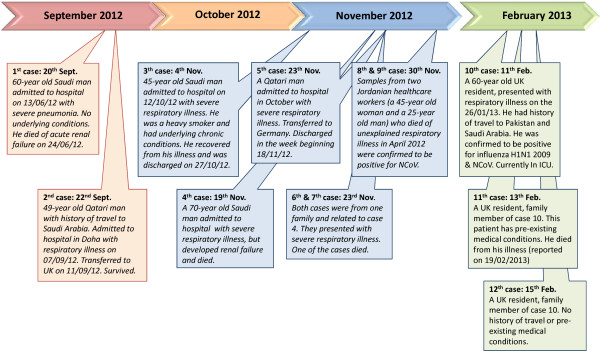
**Novel coronavirus timeline (chronology of reporting): September 2012-February 2013. **The figure summarizes some of the basic details of the 12 laboratory confirmed cases of the novel coronavirus infection and dates when these cases were officially reported.

As of 15 February 2013, a total of 12 laboratory confirmed cases of NCoV have been reported to WHO [14]. The first 9 of these cases have been from the Middle East: 5 cases (3 fatal) from Saudi Arabia, 2 cases from Qatar and 2 cases (both fatal) from Jordan (Figure 1). The most recent 3 cases (1 fatal) were in individuals living in UK.

## 20th September

The first reported case was in a 60-year old Saudi man. He was admitted to hospital in the port city of Jeddah on 13th June 2012 with a 7-day history of fever, cough and shortness of breath. He died 11-days later of progressive respiratory and renal failure [2]. Laboratory investigations for common causes of respiratory illness, including influenza, parainfluenza, adenovirus and respiratory syncytial virus were all negative [2]. However, inoculation of Vero and LLC-MK2 cells with sputum sample taken on admission, resulted in cytopathic changes suggestive of viral infection [2], which was eventually identified as a novel coronavirus and reported on 20th September [15].

## 22nd September

The second case was in a 49-year old Qatari patient [16]. He had a history of travel to Saudi Arabia from 31st July to 18th August, but no evidence of contact to the first case [16]. He developed a mild respiratory illness on 3rd of September which progressed to pneumonia and he was hospitalized in Doha on 9th September [17]. His condition further deteriorated and he was transferred by air ambulance to London. Tests for common causes of his severe respiratory illness were negative [17]. The report of the isolation of a new coronavirus from the Saudi case led the medical team to test for the new virus. The test came positive and the case was reported to the WHO on 22nd September [18].

## 4th November

No new case was reported during October. Then in early November, the Saudi Ministry of Health reported the 3rd case [19]. A 45-year old man was admitted to hospital on 12th October with severe respiratory illness. He was a heavy smoker with a number of underlying chronic conditions, including type 2 diabetes, an atrophied right kidney, and a history of ischaemic heart disease [20]. Over the next few days, his condition deteriorated with progressive pneumonia and renal failure and required mechanical ventilation and hemodialysis. As with the previous two cases, laboratory tests for common respiratory viruses were all negative [20]. However, samples tested for NCoV at three different laboratories, one in Jeddah and two in UK, all returned positive. In spite of his underlying conditions, by 27th October the patient's condition had improved and he was eventually discharged on the 4th November.

## 19th November

The fourth case was announced by the Saudi Ministry of Health on 19th November and published by ProMed-Mail two days later [21]. The patient was admitted with respiratory illness and the novel coronavirus was suspected as a possible cause. Samples were tested for the novel virus at reference laboratories in Saudi Arabia and UK and returned positive. The patient succumbed to his infection and subsequently died from renal failure [22].

## 23rd November

On 23rd November, WHO reported 3 additional confirmed cases, 1 from Qatar and 2 from Saudi Arabia [23]. The Qatari man was initially admitted to a hospital in Doha in October with severe respiratory illness, but he was subsequently transferred to a hospital in Germany. Laboratory results confirmed that he was positive for the novel coronavirus. He remained in hospital for approximately a month, but he recovered from his respiratory and renal illness and was discharged in the week of 18th November. The Saudi cases (cases 6 and 7) (Figure 1) are of particular interest as these were from a single family and were related to case 4 [22]. All 3 cases lived in the same house [24] and it is believed that case 4, a 70-year old man, was the index case in this cluster. He is thought to have infected two of his sons, one of whom (case 6) subsequently died of multi-organ failure. A fourth case from this family also presented with similar symptoms, but laboratory tests revealed that it was not due to the novel coronavirus [24].

## 30th November

At the end of November, WHO reported that two cases that had died of an unknown respiratory infection in April 2012 in Jordan were retrospectively tested for the new coronavirus and found to be positive [25]. These cases were part of a cluster of 11 cases, 8 of whom were healthcare workers who presented with a severe respiratory illness that was unexplained at the time, but was notified to the WHO [25].

## 11-15th February: family cluster

On 11th February 2013, the UK Health Protection Agency (HPA) confirmed a further case of the novel coronavirus in a 60-year old UK resident [26]. The patient was admitted to hospital on 31st January 2013 with severe lower respiratory illness. Prior to his illness, he had travelled to Pakistan (from 16th December to 20th January) and Saudi Arabia (from 20th – 28th January 2013) [27]. It appears that his illness developed while he was in Saudi Arabia. Laboratory tests confirmed NCoV infection. Interestingly, this patient was also co-infected with H1N1 2009 pandemic influenza [27]. At the time of writing this review (15th February 2013) the patient was being treated in intensive care (ICU).

A few days after the announcement of case 10, the HPA confirmed the diagnosis of two further cases, one on 13th February [28] and one of 15th February [29]. Both of these cases were family members of case 10 and neither had any recent travel history. They appear to have contracted the infection from their relative [30]. Case 11 was admitted to hospital on 9th February after a short history of respiratory symptoms. The patient had pre-existing medical conditions, which may have made him more susceptible to respiratory infections [27]. On 19th February, the HPA reported that this patient succumb to his illness and died [31] Case 12 on the other hand did not have any pre-existing medical conditions and the latest reports indicate that she has recovered. The HPA has also reported that they have identified and followed up over 100 contacts of the cases in this cluster and all have tested negative for novel coronavirus [31].

## Source and mode of transmission

The original source of infection and mode of transmission to humans is unclear. At least 2 cases were reported to have visited farms and may have had contact with animals [20,32]. Thus a zoonotic infection is a possibility. Furthermore, the fact that the NCoV is most closely related to bat coronaviruses [3-5] indicates that it might have originated from bats. Studies showing that SARS-CoV was most likely to have derived from bats [33] also supports a zoonotic origin for this new coronavirus. However, it is unknown whether the NCoV was transmitted to humans by a direct interspecies jump or it involved another intermediary animal. In the case of SARS-CoV, civet cats were identified as a likely intermediate host [8,9,33]. Like SARS-CoV, the exact natural reservoir species of the NCoV also remains to be identified.

Most of the laboratory confirmed cases of NCoV do not appear to have had any contact with animals. Could there be human-to-human transmission? The two clusters of cases, one from a family in Saudi Arabia, the other from a healthcare team at a hospital in Jordan and the most recent UK cluster of cases certainly suggests that human-to-human transmission is very likely. In the family cluster, 3 members contracted the infection. The index case is thought to be a 70-year old grandfather admitted to a hospital in Riyadh with severe respiratory illness. His two sons took in turns to stay by him during his illness (Dr Ziad Memish, personal communication). In spite of all supportive care, the patient died of renal failure. Both of his sons subsequently also contracted the infection and one of them died of multi-organ failure some days later. In the Jordanian cluster, 11 cases (7 nurses and 1 doctor) presented with an acute respiratory illness of unknown aetiology in April 2012 [25]. Two of the cases subsequently died. Retrospective testing confirmed both cases to be positive for the NCoV [34]. Investigations of the non-fatal unconfirmed probable cases in this cluster revealed that the illness in these cases was generally mild [24]. Indeed, in one case, the symptoms were mild enough to be managed at home. The cases in both, the Saudi and Jordanian cluster had no reported contact with animals. Similarly, the most recent laboratory confirmed cases from UK (cases 11 and 12), also had no contact with animals or any history of travel to the Middle East. Both appear to have contracted the infection from a family member who had recently returned from Saudi Arabia [30]. Together, these anecdotal observations clearly indicate that person-to-person infection, probably via respiratory mode of transmission, is highly plausible.

Based on the data we have so far, NCoV does not appear to be highly infectious in humans. None of the first 3 confirmed cases (2 Saudis, 1 Qatari) have been associated with transmission of symptomatic disease to close contacts [20,32]. Detailed follow-up of 64 close contacts of the Qatari patient, which included healthcare professionals, family members and friends, only 13 individuals developed respiratory symptoms, but none of these cases were positive for NCoV [32]. This would suggest that human-to-human transmission is limited.

## Clinical features associated with the novel coronavirus

All of the cases had one thing in common: they suffered from severe respiratory illness which was not due to any of the known viral or bacterial causes. The most common initial symptoms were reported to be fever, cough and shortness of breath. Patients rapidly progressed to severe pneumonia and renal failure. The latter presentation has not been seen in all patients. For examples, none of the cases in the Jordanian cluster had renal failure [24]. The two fatal cases in this cluster, one developed pericarditis and the other had disseminated intravascular coagulation [24]. Coronaviruses predominantly cause mild self-limiting upper respiratory tract infections. The only other human coronavirus that is associated with severe lower respiratory infection is SARS-CoV [35]. However, in contrast to SARS-CoV [36,37], this novel coronavirus does not appear to cause diarrhea. Of 12 laboratory confirmed cases, 6 have died and 1 is currently in ICU. This would imply a relatively high mortality rate. However, caution has to be exercised, since we do not know the true prevalence of infection with NCoV. It is possible that in some cases, the virus is associated with mild respiratory tract infection which goes unseen and only those patients who develop severe disease seek medical attention. It is also worth noting that all of laboratory confirmed cases have been adults.

## Diagnosis of the novel coronavirus

The novel coronavirus can be cultured from sputum samples using monkey kidney cells, Vero and LLC-MK2 cells. Viral induced cytopathic changes are seen in these cells within 1–2 weeks of infection [2]. However, these changes are not specific for NCoV and confirmation using reverse transcription PCR (RT-PCR) is required. RT-PCR can also be performed directly on clinical samples such as respiratory swabs. An optimized real-time RT-PCR protocol for the specific detection of NCoV has been developed and is available [1]. Furthermore, an additional confirmatory RT-PCR assay and a serological test using convalescent patient serum has been established [38].

## Concluding remarks

The emergence of any novel virus, in particular one that can be transmitted via the respiratory route, has to be taken seriously. A rapid response, a united global front and mobilization of resources and expertise are our best tools in preventing or reducing the devastation that some of these viruses can cause. This is exemplified by the recent events that have led to the recognition and isolation of the novel coronavirus responsible for acute respiratory illness. Within weeks of the first report, a PCR-based diagnostic assay was made available [1], a preliminary case definition [39] and incubation period was issued [40], the virus was fully sequenced [4], the detection of new cases was rapidly communicated to the health authorities and more recently, guidelines for handling and working with this virus have been issued [24]. The novel coronavirus is the 6th member of the human coronaviruses and the third to be isolated in the last ten years. Based on current information, NCoV does not appear to transmit easily or sustainably between people, but it can lead to serious lower respiratory tract infection and renal failure. The mode of transmission has not been conclusively identified, although respiratory route looks most likely. A study which is in press, has shown that a number of different bat species resident in Ghana and parts of Europe are infected with coronaviruses very similar to NCoV, in some cases differing by less than 2% at the genetic level [41]. These findings suggest that NCoV is most likely to have arisen from bat viruses. However, a number of pertinent questions remain unanswered: Does NCoV represent an interspecies jump of a bat coronavirus? How did humans acquired the infection? Was it a direct infection from bats to humans or did it involve an intermediate host such as domestic animals? Are the virus isolates from all infected persons genetically identical? Do NCoV genetic variants exits in the human population, but cause mild or asymptomatic infections? Future studies will no doubt attempt to address these and other related questions. For the time being, surveillance and thorough investigations of cases with unexplained severe respiratory illness, particularly in those residing in or returning from Middle East, is being recommended [24].

## Competing interest

The author declare that he had no competing interests.

## Note added in proof

On 21^st^ of February 2013, another confirmed case was reported from Saudi Arabia. The patient was hospitalized on 31^st^ January and died on 10^th^ February.
